# Maternal mental health and its intergenerational transmission in-utero: a scoping review

**DOI:** 10.1080/16549716.2026.2750711

**Published:** 2026-10-22

**Authors:** Minnat Seema, Clifford Afoakwah, Joshua Byrnes

**Affiliations:** a Centre for Applied Health Economics (CAHE), Griffith University, Brisbane, Australia; b Australian Centre for Health Services Innovation, School of Public Health and Social Work, Queensland University of Technology, Brisbane, Australia; c Jamieson Trauma Institute, Metro North Health, Brisbane, Australia

**Keywords:** Maternal mental health, pregnancy, in-utero transmission, intergenerational health, developmental origins

## Abstract

Maternal mental health during pregnancy is important for maternal wellbeing and offspring development. Prenatal depression, anxiety, stress, and psychological distress have been associated with offspring emotional, behavioural, cognitive, and mental health outcomes, but evidence remains dispersed across conditions, outcomes, and proposed mechanisms. This scoping review synthesises empirical evidence on maternal mental health during pregnancy as a potential intergenerational exposure, including potential in-utero pathways.

Five databases (MEDLINE, Scopus, PsycInfo, CINAHL, and Embase) were searched from 1995 to 24 February 2026, following PRISMA-ScR guidance. Eligible sources of evidence examined maternal mental health during pregnancy in relation to postnatal offspring outcomes. Data were extracted using a structured framework and synthesised narratively. Twenty sources of evidence met the inclusion criteria. Across diverse designs and populations, prenatal depression, anxiety, stress, and psychological distress were commonly associated with adverse offspring outcomes, including emotional and behavioural difficulties, neurocognitive impairments, and increased risk of psychiatric conditions from childhood to adulthood. Several primary studies reported associations independent of traditional birth outcomes, consistent with potential in-utero pathways. Plausible mechanisms included stress-related neuroendocrine processes, inflammation, and epigenetic regulation. However, heterogeneity in exposure measurement, outcome definitions, and follow-up periods limited comparability and causal interpretation.

Maternal mental health during pregnancy may be an important early-life and intergenerational exposure associated with offspring developmental trajectories, but observational evidence does not establish causal in-utero transmission. Stronger causal inference, longitudinal data linkage, and incorporation of economic and policy-relevant outcomes are priorities for future research.

## Paper Context

**Main findings**: Prenatal maternal mental health problems, including depression, anxiety, and stress, are commonly associated with adverse offspring emotional, behavioural, and neurocognitive outcomes across the life course.

**Added knowledge**: This review conceptualises maternal mental health during pregnancy as a potential intergenerational exposure, including potential in-utero pathways, and identifies key gaps in causal evidence, measurement consistency, and longitudinal research.

**Global health impact for policy and action**: Recognising maternal mental health during pregnancy as an important early-life exposure may inform preventive strategies and strengthen global health policies aimed at improving long-term developmental and population health outcomes.

## Background

Mental health disorders constitute a leading and persistent source of global disability across the life course. In 2019, mental disorders accounted for an estimated 125.3 million years lived with disability globally, representing 14.6% of all years lived with disability [1]. According to the World Health Organisation, mental health conditions are projected to remain among the leading causes of disability worldwide, imposing substantial health and economic costs on individuals and healthcare systems [2]. Perinatal mental health conditions are also common, with approximately one in five women experiencing a mental health condition during pregnancy or within the first year after birth, and the burden is disproportionately higher in low- and middle-income countries than in high-income countries [3]. Among vulnerable populations, pregnant women face particular challenges, as maternal mental health disorders, especially depression, anxiety, and stress, have been associated with adverse maternal and foetal outcomes, including pregnancy complications, compromised maternal health, and long-term implications for child development [4–6]. The global pooled prevalence of perinatal depression has been estimated at 11.9%, while anxiety symptoms affect approximately 18.2%, 19.1%, and 24.6% of women during the first, second, and third trimesters of pregnancy, respectively [7,8]. Pregnancy represents a critical developmental window marked by substantial physiological and psychosocial change, with important consequences for both maternal health trajectories and offspring development [9,10].

Mental health during pregnancy has long been recognised as a key determinant of maternal wellbeing [11]. Far less attention, however, has been paid to its role as a potential intergenerational exposure transmitted in utero, with potential consequences extending beyond infancy and across the child’s life course [12]. This gap is notable given accumulating evidence that prenatal psychological distress, depression, and anxiety are associated with offspring mental health, cognitive development, and emotional functioning [13–15]. Previous reviews have similarly reported associations between antenatal maternal stress and anxiety and later offspring neurodevelopmental, behavioural, and mental health outcomes, while emphasising that the underlying mechanisms and causal pathways remain incompletely understood [16,17]. These associations may reflect biological and psychosocial processes operating during gestation, including stress-related neuroendocrine processes, inflammatory responses, and alterations in foetal neurodevelopment, alongside maternal behaviours and contextual factors that co-occur with prenatal distress.

Despite growing interest, the literature remains fragmented across disciplines, with substantial heterogeneity in exposure definitions, timing of assessment, outcome measurement, and follow-up periods [13,18,19]. As a result, prenatal maternal mental health is rarely conceptualised systematically as an intergenerational determinant of health. This limitation may lead to underinvestment in prevention and biased estimates of the long-term returns to maternal and early-childhood interventions [20,21].

This gap has important implications. If maternal mental health during pregnancy operates as a persistent intergenerational exposure, its omission from policy and economic frameworks represents a missed opportunity for early prevention [22,23]. Understanding when, how, and under what circumstances prenatal mental health influences offspring outcomes is therefore essential for informing maternal mental health policy, early childhood investment, and broader strategies aimed at reducing intergenerational disadvantage [24,25].

The present review adopts a global perspective because maternal mental health is a universal public health concern, while the biological and developmental processes through which prenatal mental health may influence offspring outcomes are relevant across diverse healthcare systems and socioeconomic settings [24,25]. The review is particularly timely given increasing recognition of the Developmental Origins of Health and Disease framework, growing policy emphasis on early-life interventions, and renewed global attention to integrating perinatal mental health into maternal and child health services [3,26].

Although previous reviews have examined specific aspects of prenatal maternal mental health, including depression, anxiety, stress, or individual biological mechanisms [27–30], few have synthesised this evidence within a unified intergenerational framework. Existing reviews have generally focused on clinical or developmental outcomes without explicitly considering the broader implications for population health, health economics, and policy [12,28–31]. This review extends the existing literature by conceptualising maternal mental health during pregnancy as an intergenerational in-utero exposure, integrating evidence across diverse mental health conditions, developmental outcomes, and proposed biological pathways, while identifying conceptual and methodological gaps to inform future research and policy.

Against this background, this scoping review aims to (i) map the empirical literature examining maternal mental health during pregnancy as a prenatal exposure, (ii) characterise study designs, exposure measures, timing, and offspring outcomes, and (iii) identify conceptual and methodological gaps relevant to research on maternal mental health and offspring outcomes.

Figure 1 presents the conceptual framework guiding this review, illustrating how prenatal maternal mental health shapes the foetal environment through in-utero pathways and influences offspring outcomes across the life course, with broader economic and social implications.

**Figure 1.**
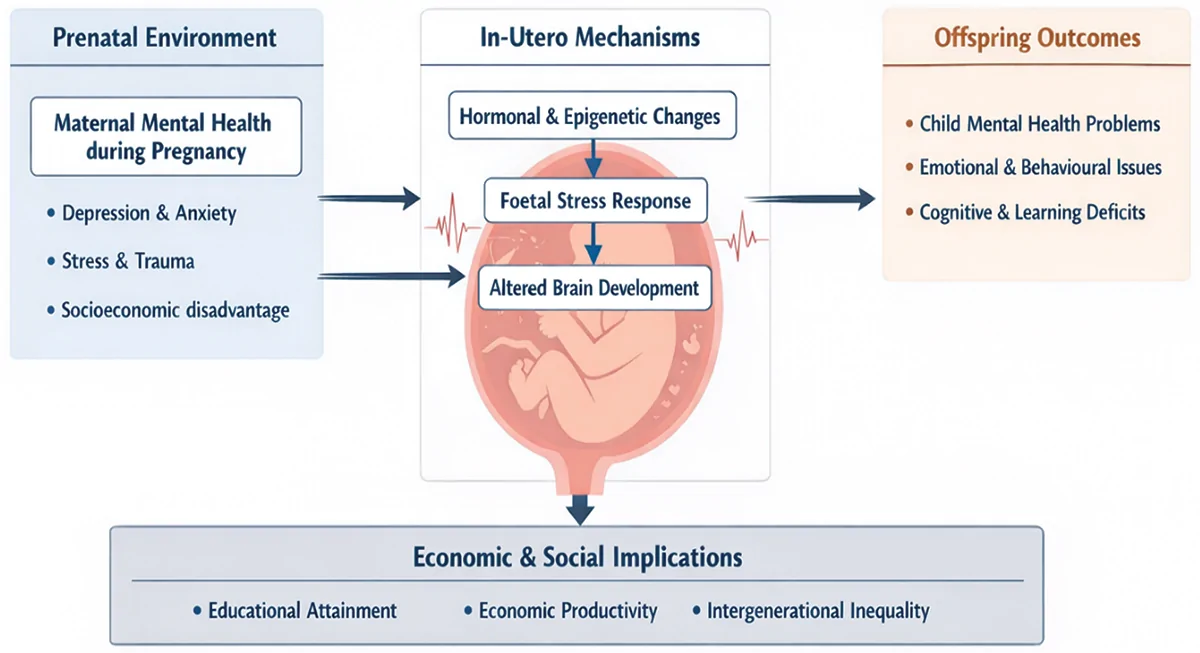
In-utero transmission as an intergenerational health shock.

Conceptual framework illustrating how prenatal maternal mental health influences offspring outcomes through biological pathways and broader economic and social implications.

## Methods

### Study design and reporting framework

This study was conducted as a scoping review to map the existing evidence on maternal mental health during pregnancy and its potential in-utero pathways to offspring mental, cognitive, and psychological outcomes. The review was designed and reported in accordance with the PRISMA Extension for Scoping Reviews (PRISMA-ScR) guidelines (Supplementary File 1).

A scoping review was selected in preference to a systematic review because the primary objective was to comprehensively map and conceptualise the breadth of evidence, rather than estimate pooled effect sizes or evaluate intervention effectiveness. The literature spans multiple disciplines and is characterised by substantial heterogeneity in study designs, exposure definitions, measurement instruments, offspring outcomes, follow-up periods, and proposed biological mechanisms. Given this diversity, a scoping review was considered the most appropriate methodology to synthesise the available evidence, identify conceptual and methodological gaps, and inform future research and policy.

### Eligibility criteria and conceptual framework

The review was guided by a PECO-informed framework appropriate for observational exposure-based research. Sources of evidence were eligible for inclusion if they involved pregnant women and their offspring, and examined maternal mental health assessed during pregnancy as the exposure of interest. Maternal mental health was conceptualised as a multidimensional exposure domain encompassing depression, anxiety, psychological distress, stress, and trauma-related symptoms, as measured using validated instruments. Comparator groups were not mandatory and varied across primary studies. Eligible sources of evidence reported postnatal offspring mental health, emotional and behavioural outcomes, cognitive development, or psychological wellbeing assessed beyond birth.

### Exclusion criteria

Sources of evidence were excluded if they focused exclusively on perinatal outcomes (e.g. birth weight, gestational age, and Apgar scores) without relevance to longer-term developmental outcomes. Conference abstracts, editorials, commentaries, protocols, dissertations, grey literature, and non-English publications were also excluded. Sources of evidence incorporating broader maternal mental health trajectories (e.g. preconception or postnatal exposures) were retained only where maternal mental health during pregnancy was measured as a distinct exposure or where the findings enabled prenatal associations to be distinguished from preconception or postnatal influences. Information on mental health before pregnancy or after birth was used to contextualise developmental pathways and possible confounding or postnatal influences; it was not treated as direct evidence of a prenatal association where pregnancy-specific findings were not reported.

### Information sources and search strategy

Five electronic databases (MEDLINE, Scopus, PsycInfo, CINAHL, and Embase) were searched from 1995 to 24 February 2026. The 1995 start date was selected pragmatically to focus the review on the contemporary era of perinatal mental health research, during which validated mental health assessment tools and prospective cohort designs became more widely used. Earlier literature was excluded to maintain a methodologically and conceptually comparable evidence base and to enable feasible mapping of a large, multidisciplinary literature. The search strategy combined controlled vocabulary (e.g. Medical Subject Headings [MeSH], where applicable) and free-text terms using Boolean operators (AND/OR). The three principal search concepts were:

- pregnancy and the prenatal period (e.g. *pregnancy*, *pregnant women*, *prenatal*, *antenatal*, *gestation*)
- maternal mental health (e.g. *maternal mental health*, *depression*, *anxiety*, *psychological distress*, *stress*, *mental disorders*); and
- offspring outcomes and intergenerational transmission (e.g. *offspring*, *child development*, *mental health*, *cognitive development*, *behaviour*, *intergenerational*, *foetal programming*).

The three search concepts were combined using the Boolean operator AND, while related terms within each concept were combined using OR.

Searches were limited to peer-reviewed journal articles published in English due to feasibility considerations and to ensure consistency in measurement frameworks. In addition, the reference lists of included sources of evidence were manually screened to identify additional relevant articles that may not have been captured in the database searches.

The complete database-specific search strategies are provided in the Supplementary Document (S1–S5).

### Study selection

The database search yielded 6,930 records (MEDLINE: 1,665 (S1); Scopus: 1,970 (S2); PsycInfo: 60 (S3); CINAHL: 589 (S4); Embase: 2,646 (S5)). After removal of duplicates, 3,845 unique records were retained for screening.

Study selection followed a staged process consistent with scoping review methodology. Two reviewers (MS and CA) independently screened titles and abstracts using the predefined eligibility criteria. Any disagreements were resolved through discussion and, where consensus could not be reached, through consultation with a third reviewer (JB). Full-text screening was subsequently undertaken using the same predefined eligibility criteria.

Following title and abstract screening of 1,875 records, 50 articles underwent full-text review assessment. Of these, 20 sources of evidence met all inclusion criteria and were included in the final synthesis as shown in Figure 2.

**Figure 2.**
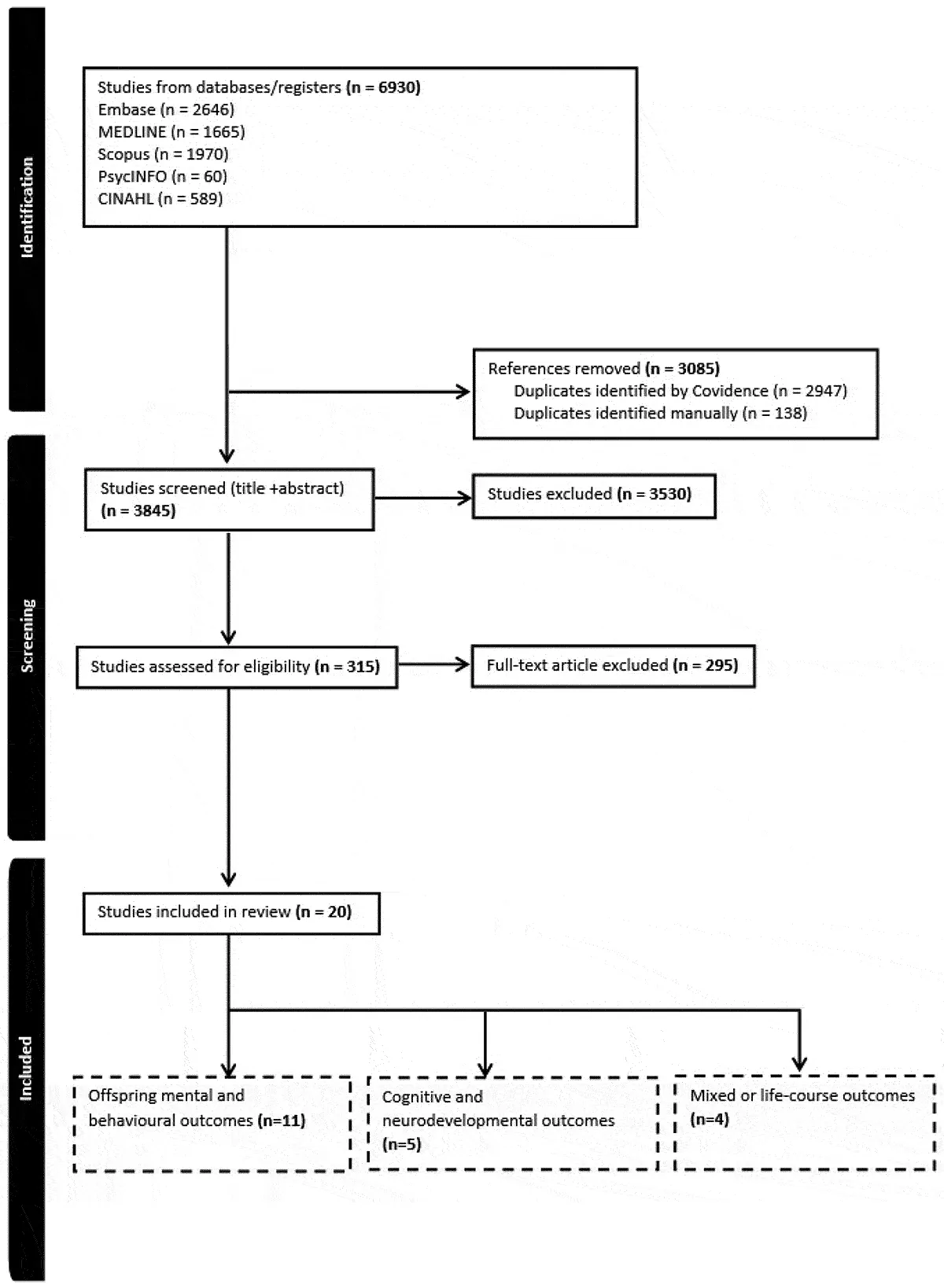
PRISMA-ScR flow diagram.

Flow diagram showing study identification, screening, eligibility, and inclusion process.

### Data charting, synthesis, and critical appraisal

Data were charted using a structured extraction framework capturing study characteristics, exposure measures, timing of exposure, conceptualisation of in-utero pathways, offspring outcomes, and key findings.

The included sources of evidence comprised primary empirical studies, systematic reviews, meta-analyses, and narrative reviews. Potential overlap between primary studies and synthesis-based evidence was considered during charting. Each source was classified by evidence type and treated as a separate source of evidence; review-level findings were used to contextualise and compare the literature rather than as additional independent observations. No quantitative pooling or counting of findings across primary and secondary sources was undertaken. Consequently, where similar findings appeared in both a primary study and a review, they were interpreted as corroborative rather than as separate evidence of effect.

Given the heterogeneity in study designs, exposure definitions, and outcome measures, findings were synthesised narratively using a thematic approach. Sources of evidence were grouped according to maternal mental health exposures, offspring outcome domains, and proposed biological pathways to identify common themes, patterns, and evidence gaps across the literature. Consistent with PRISMA-ScR guidance, no formal risk-of-bias assessment was undertaken, as the primary objective of this review was to map the extent, nature, and characteristics of the available evidence rather than to evaluate study quality or estimate causal effects.

## Results

### Characteristics of included evidence

Twenty sources of evidence were included (Table 1), comprising prospective birth cohorts, register-based analyses, neuroimaging follow-ups, and synthesis-based evidence examining prenatal maternal mental health and offspring outcomes.

**Table 1.** Characteristics of included sources of evidence.

Author (Year)	Country / Cohort	Study Design	Prenatal Maternal Mental Health Exposure	Timing of Exposure	Offspring Outcome Domain	Follow-up Period
Van den Bergh et al. [32]	Multiple international prospective cohorts (primarily Europe and North America)	Narrative review of prospective cohort and foetal studies	Antenatal anxiety and stress (including state, trait and pregnancy-specific anxiety; some inclusion of depression)	Across pregnancy; foetal effects observable from ~27–28 weeks’ gestation	Foetal neurobehaviour; cognitive development; behavioural and emotional problems; attentional difficulties	Foetal period to adolescence (up to approximately 14–15 years in included cohorts)
Walsh et al. [33]	United States; predominantly Latina pregnant adolescents	Prospective cohort study	Prenatal depressive symptoms; interaction with maternal history of childhood abuse	Second and third trimester; biomarkers measured at 24–27 and 34–37 weeks’ gestation	Birth outcomes (gestational age, birth weight, pregnancy complications)	Birth (delivery outcomes)
Wei et al. [34]	Singapore; Growing Up in Singapore Towards Healthy Outcomes (GUSTO) birth cohort	Prospective longitudinal birth cohort with repeated neuroimaging	Prenatal maternal depressive symptoms (EPDS)	26 weeks’ gestation (second trimester)	Cortical morphology; reward sensitivity	
Pariante [35]	United Kingdom; review integrating human, animal and cellular studies	Narrative molecular review	Antenatal (prenatal) depression (major depressive disorder during pregnancy)	Across pregnancy (focus on biological alterations during gestation; no specific gestational week defined)	Maternal behaviour; offspring emotional and behavioural problems; stress reactivity; epigenetic regulation	Foetal period to adolescence (based on synthesised human and animal evidence)
Spry et al. [36]	Australia – Victorian Intergenerational Health Cohort Study (VIHCS) & Australian Temperament Project Generation 3 (ATPG3)	Prospective intergenerational cohort (two-cohort integrated dataset)	Persistent maternal mental health problems (adolescent & young adulthood); antenatal depressive symptoms; postnatal depressive symptoms	Preconception (adolescence & young adulthood); Third trimester; 1 year postpartum	Infant emotional reactivity (temperament)	Infant assessed at 1 year postpartum
Davis & Narayan [37]	Multiple (international literature synthesis)	Narrative review	Prenatal stress; depression; anxiety; PTSD; maternal adversity (childhood & adulthood)	Preconception and pregnancy (across trimesters; life-course perspective)	Birth outcomes; infant stress regulation; emotional development; cognitive development; internalising psychopathology; HPA-axis functioning	
O’Donnell et al. [38]	United Kingdom – Avon Longitudinal Study of Parents and Children (ALSPAC)	Prospective population-based longitudinal cohort	Maternal prenatal anxiety (Crown–Crisp Experiential Index); prenatal depression (EPDS)	18 weeks and 32 weeks gestation	Child emotional and behavioural problems (SDQ: total, emotional, conduct, ADHD); probable mental disorder	Ages 4, 7, 9, 11.5, and 13 years
Lewis et al. [26]	Australia (institutional affiliations; review of international evidence)	Narrative review / conceptual synthesis (DOHaD framework)	Maternal depression, anxiety, stress; lifestyle factors; teratogenic and neurotoxic exposures	Preconception, pregnancy, early postnatal period (first 1,000 days)	Child and adolescent mental disorders (emotional, behavioural, hyperactivity disorders); HPA-axis dysregulation; neurodevelopmental vulnerability	Not applicable (review paper synthesising longitudinal and epidemiological studies)
Robinson et al. [28]	Multi-country (Finland, UK, Netherlands, Germany, Norway, USA – synthesis of international cohorts including ALSPAC, PREDO, Generation R, MoBa, SLCD, registry data)	Systematic mini review (13 empirical studies; longitudinal and cohort designs)	Clinically diagnosed depression or depressive symptoms during pregnancy	Assessed across pregnancy (varied by study; some multi-timepoint, e.g. bi-weekly in PREDO; others single trimester assessment)	Neuropsychiatric outcomes: internalising, externalising, ADHD, autism spectrum disorder, anxiety, depression, behavioural problems	Follow-up ranged from toddlerhood (2 years) to adolescence (18 years) and adulthood (25 years), depending on cohort
Bleker et al. [27]	Multi-country (USA, UK, Canada, China, India; synthesis of 13 prospective cohorts)	Systematic review of prospective cohort studies	Prenatal depressive disorder or depressive symptoms (various validated scales, e.g. EPDS, BDI, CES-D, DSM-IV interviews)	Assessed across pregnancy (varied by study; mostly second and third trimester)	HPA-axis reactivity (salivary cortisol); autonomic nervous system reactivity (heart rate, blood pressure, respiratory sinus arrhythmia, salivary alpha-amylase)	Offspring assessed from infancy (1–7 months) to adolescence (15 years), depending on study
Turk et al. [39]	Belgium; Prospective Prenatal Longitudinal Study (PELS cohort)	Prospective cohort with 28-year follow-up; resting-state fMRI study	Maternal state anxiety (self-reported)	12–22 weeks gestation (second trimester)	Adult brain functional connectivity (prefrontal networks)	28 years (adulthood)
Chae et al. [40]	Chile; Santiago Longitudinal Study (SLS)	Prospective longitudinal cohort with growth mixture modelling	Maternal depressive symptoms (CES-D)	Assessed when child was 1, 5.5, 10, and 14.6 years (postnatal trajectory; not prenatal-specific)	Adolescent and young adult psychosocial adjustment (internalising, externalising, attention); adolescent cognitive functioning (verbal, reading, mathematics)	Infancy → adolescence (14–17 years) → young adulthood (20–23 years)
Haavik et al. [41]	Norway (multigenerational family data referenced) + International literature synthesis	Perspective / narrative review (with reference to prior human family-based genetic study)	Maternal genetic determinants of neurobiological pathways influencing mental health (e.g. TPH1 mutations affecting serotonin synthesis; thyroid hormone, folate metabolism, immune and glucocorticoid-related genes)	Prenatal (in-utero genetic programming mechanisms)	ADHD symptoms; neurodevelopmental disorders (ADHD, schizophrenia, autism); cognitive impairment	Not applicable (conceptual review; referenced family study included cross-generational data)
Waxler et al. [42]	Multiple countries (literature synthesis)	Narrative review	Perinatal maternal depression (antenatal and postpartum)	Second and third trimester highlighted as critical prenatal window; also, postpartum period	Foetal growth; preterm birth; infant temperament; HPA-axis regulation; attachment; later behavioural and emotional problems	Not applicable (review synthesising short- and long-term outcomes from infancy to adolescence)
Glover [43]	International (literature synthesis)	Narrative review	Prenatal maternal depression, anxiety, and stress (including disaster exposure, bereavement, relationship stress, pregnancy-specific anxiety)	Across gestation, trimester-specific sensitivity discussed (first trimester for schizophrenia; later pregnancy for conduct disorder)	Emotional and behavioural problems; ADHD; cognitive development; birthweight; gestational age; HPA-axis function; telomere length; brain structure	Not applicable (review synthesising outcomes from birth to adulthood)
Stürmlinger et al. [44]	Germany / German Family Panel (pairfam)	Prospective longitudinal cohort	Prenatal depressive symptoms (State–Trait Depression Scales)	Pregnancy (T0)	Child adjustment difficulties (internalising & externalising behaviours via SDQ)	From pregnancy to age 3 years
Tuovinen et al. [30]	Review of studies conducted in Europe & Canada (e.g. PREDO, Generation R, EDEN, Project Viva, Rhea)	Narrative review (human prospective cohort & case-control studies, 2017–2019)	Maternal prenatal depression, stress symptoms, mood disorders	Pregnancy (varied by study; typically, mid–late gestation)	Child neurocognitive outcomes (IQ, language, motor skills, developmental milestones, executive function)	Infancy to middle childhood (6 months to 8 years across included studies)
Entringer et al. [45]	USA / Germany (conceptual & empirical synthesis)	Narrative review / psychobiological synthesis	Maternal psychological stress, anxiety, depression, stress biology (cortisol, inflammatory markers)	Pregnancy (early-late gestation)	Neurodevelopment, metabolic function, immune function, telomere biology, disease susceptibility	Across lifespan (newborn to adulthood; synthesis of multiple cohorts)
Kim et al. [46]	South Africa – Birth to Twenty Plus (Bt20+), Soweto–Johannesburg	Prospective longitudinal birth cohort	Prenatal stress (16-item scale assessing psychosocial adversity, violence, trauma during pregnancy under apartheid)	Third trimester (stress reported for previous 6 months during pregnancy)	Psychiatric morbidity (GHQ-28), stress sensitisation effects	17–18 years (late adolescence / early adulthood)
Tirumalaraju et al. [29]	Multiple countries (UK, USA, Australia, Finland cohorts included in meta-analysis)	Systematic review and meta-analysis of prospective longitudinal cohort studies	Maternal perinatal depression (antenatal and/or postnatal depressive symptoms; assessed via validated scales/interviews such as EPDS, CIS, CES-D, etc.)	Antenatal (during pregnancy; various trimesters) and/or postnatal (within 12 months postpartum)	Offspring depression (adolescent and adult depressive disorders assessed using standardised scales or diagnostic interviews)	Offspring assessed ≥ 12 years; follow-up ranged from adolescence (~16 years) to adulthood (up to 43 years)

Sources of evidence were geographically diverse, including low- and middle-income countries (*n* = 8), the United States (*n* = 2), the United Kingdom (*n* = 2), Australia (*n* = 2), and several other countries (*n* = 1 each), with follow-up periods ranging from early childhood to adulthood.

Prenatal exposures included maternal depressive symptoms, anxiety, psychological distress, trauma-related stress, and biological stress markers. Most primary studies assessed exposures during the second and/or third trimester, reflecting key periods of foetal neurodevelopment. A small number of sources included preconception or postnatal maternal mental health measures in addition to pregnancy assessments. These sources were retained where prenatal findings could be distinguished and were used to contextualise developmental pathways and the potential influence of non-prenatal factors.

Overall, the included sources of evidence comprised a diverse range of study designs and evidence types, as summarised in Table 2.

**Table 2.** Summary of the methodological characteristics of included sources of evidence (*n* = 20).

Study design	Number
Prospective cohort / longitudinal cohort	7
Birth cohort	2
Narrative reviews	6
Systematic reviews / meta-analyses	3
Conceptual / perspective papers	2

Note: Evidence types were classified according to the primary methodological approach reported by each included source.

### Prenatal maternal depression and offspring outcomes across development

Across both primary empirical studies and synthesis-based evidence, prenatal maternal depression was commonly associated with adverse offspring outcomes spanning behavioural, emotional, cognitive, and psychiatric domains (Table 2).

Longitudinal studies report that higher antenatal depressive symptoms are associated with poorer early childhood adjustment (B = 0.018, 95% CI: 0.008–0.026; β = 0.227; *p* < .001), while reductions in maternal symptoms are associated with improved outcomes (B = −0.019, 95% CI: −0.027 to −0.011; β = −0.248; *p* < .001) [44].

At the biological level, several primary studies report associations between maternal depression and inflammatory markers (interleukin-6: B = 0.006, *p* = .011), gestational processes (B = 0.004, *p* = .040), and cortisol-related pathways (β = −0.143, *p* < .039), suggesting involvement of hypothalamic–pituitary–adrenal (HPA) axis functioning [33]. Evidence from systematic reviews further describes potential roles for inflammation, epigenetic regulation, and neuroendocrine processes [28].

At the population level, antenatal distress is associated with increased risks of adverse developmental outcomes (e.g. OR = 8.4, 95% CI: 2.4–29.0; OR = 3.6, 95% CI: 1.3–10.3), although effect sizes vary across studies [32]. Chronic maternal depression appears to be associated with stronger effects on offspring internalising (β = 0.24, *p* < .01) and externalising behaviours (β = 0.21, *p* < .01), while declining symptom trajectories are associated with attenuated associations [40].

These associations extend into later life. Meta-analytic evidence indicates an increased risk of depression among exposed offspring (OR = 1.70, 95% CrI: 1.60–2.65), with individual primary studies reporting 1.3- to 4.7-fold increases [29]. Despite variability in magnitude, the direction of findings is broadly consistent across studies.

### Prenatal anxiety, stress and neurodevelopmental programming

Prenatal anxiety and stress were also frequently examined across the included source of evidence. Evidence from prospective cohorts indicates associations with adverse cognitive, behavioural, and emotional outcomes, in some cases independent of postnatal maternal mental health.

Reported associations include increased risks of developmental abnormalities (OR = 3.6, 95% CI: 1.3–10.3) and, in some studies, congenital outcomes (OR = 8.4, 95% CI: 2.4–29.0), although findings are heterogeneous [32].

Some studies suggest that vulnerability may precede pregnancy. Preconception maternal mental health problems have been associated with higher infant emotional reactivity (37% vs 23%; OR = 2.1, 95% CI: 1.4–3.1), with a substantial proportion of the association not explained by antenatal or postnatal exposures [36].

Neuroimaging evidence indicates associations between prenatal anxiety and altered brain development, including reduced prefrontal functional connectivity in adult offspring (*p* < 0.05) [39], and structural differences in cortical regions linked to behavioural outcomes such as reward sensitivity (*p* < 0.05) [34].

Interaction analyses further suggest that prenatal stress may modify responses to later-life adversity, with stronger associations observed when prenatal and postnatal stressors co-occur (b = 1.19, *p* = .034) and variation by maternal age (b = −0.17, *p* = .008) [46].

### Adult neurobiological and psychiatric persistence

Evidence indicates that associations between prenatal maternal mental health and offspring outcomes extend into adolescence and adulthood.

A meta-analysis of six cohort studies (*n* = 15,584) reports increased risk of depression among exposed offspring (OR = 1.70, 95% CrI: 1.60–2.65), with similar antenatal-specific estimates (OR = 1.78, 95% CrI: 0.93–3.33) and limited variation by sex (OR = 1.06, 95% CrI: 0.99–1.14) [29].

Neuroimaging studies report persistent differences in brain function, including reduced prefrontal connectivity in adult offspring exposed to prenatal anxiety (*p* < 0.05) [39].

Overall, findings across longitudinal and neurobiological studies indicate that associations observed in early life may persist into later developmental stages, although the extent to which these reflect prenatal versus postnatal influences varies across studies.

### Potential biological pathways linking prenatal maternal mental health and offspring outcomes

Evidence points to several plausible biological pathways that may link prenatal maternal mental health with offspring outcomes, including neuroendocrine, immune, genetic, and epigenetic processes. Maternal stress has been associated with altered cortisol levels, placental function, and foetal glucocorticoid exposure. Some studies have reported that cortisol-related processes account for variation in gestational age and foetal growth, and that exposed newborns may show altered cortisol levels [42].

Epigenetic processes, including altered methylation of glucocorticoid receptor genes, immune pathways and brain differences such as reduced grey matter volume have also been proposed as plausible mechanisms [35,45].

However, findings are not uniform. For example, only 3 of 13 studies report significant associations between prenatal depression and offspring stress reactivity [27], and several studies indicate attenuation after adjustment for postnatal environments. Maternal depression is also associated with parenting and environmental factors that may influence offspring outcomes [42].

Emerging genetic evidence further suggests gene–environment interplay, with maternal genetic liability contributing to both intrauterine conditions and offspring outcomes [41].

### Neurocognitive outcomes

Evidence on neurocognitive outcomes is mixed. Neuroimaging studies report associations between prenatal maternal depressive symptoms and reduced cortical thickness in prefrontal regions, with indirect associations with behavioural outcomes such as reward sensitivity (*p* < 0.05) [34].

At the population level, prenatal depression and anxiety, affecting approximately 10–13% of women (up to 20–30% including subthreshold symptoms), have been associated with lower cognitive performance, including reductions in verbal IQ (B ≈ −5.06) and full-scale IQ (B ≈ −7.46). However, these associations are frequently attenuated after adjustment for socio-demographic and postnatal factors.

Some studies report persistent associations. For example, one cohort (*n* = 2,231) found lower developmental scores (B = −0.84, 95% CI: −1.13 to −0.55) and increased developmental delay (OR = 1.33, 95% CI: 1.17–1.50) [30], while others report null or subgroup-specific findings.

Overall, findings suggest potential associations between prenatal maternal mental health and neurocognitive development, although effect sizes are modest and heterogeneity across studies is substantial.

### Overall synthesis

Across the included evidence, several consistent patterns emerge.

First, prenatal maternal mental health, particularly depression, anxiety, and stress, is commonly associated with offspring behavioural, emotional, and psychiatric outcomes across the life course. These associations are observed from early childhood through adolescence and, in some studies, into adulthood [17,29,30,40,44].

Second, evidence from longitudinal, neuroimaging, and synthesis studies suggests that these associations may operate through multiple pathways, including neuroendocrine processes, inflammation, and epigenetic regulation, alongside structural and functional brain differences [28,33–35,39,42]

Third, the magnitude and persistence of associations appear to vary across contexts, with several studies reporting attenuation after adjustment for socio-demographic and postnatal factors, indicating the importance of environmental influences [17,27].

Finally, some evidence suggests that prenatal exposure may modify vulnerability to later-life stressors, consistent with a stress-sensitisation framework [46].

Overall, the literature is consistent with a life-course perspective in which prenatal maternal mental health represents an early exposure linked to later developmental trajectories, although heterogeneity in findings highlights ongoing uncertainty regarding causal pathways and the relative contribution of prenatal and postnatal factors.

## Discussion

### Principal findings

This scoping review mapped the empirical evidence on maternal mental health during pregnancy and its association with offspring mental, cognitive, and psychological outcomes, as summarised in Table 3. Across the included sources of evidence, prenatal exposure to maternal depression, anxiety, stress, and psychological distress was commonly associated with adverse offspring outcomes extending beyond birth and infancy. Importantly, several primary studies reported associations independent of traditional birth outcomes, suggesting that the observed relationships may not operate solely through foetal growth restriction or perinatal complications. Collectively, the evidence indicates that maternal mental health during pregnancy may represent an important early-life exposure with implications extending across childhood and adolescence.

**Table 3.** Relevant evidence on prenatal maternal mental health and in-utero intergenerational transmission.

Author (Year)	Study Design / Cohort	Prenatal Maternal Mental Health Exposure	Timing / In-utero Pathway	Offspring Outcome(s)	Key Results Relevant to Review Question
Van den Bergh et al. [32]	Narrative synthesis of prospective cohorts	Antenatal anxiety and stress	Proposed foetal programming via placental cortisol transfer and altered HPA-axis functioning	Foetal neurobehaviour; child behavioural and emotional problems	Consistent evidence that antenatal anxiety is associated with altered foetal heart rate variability and increased behavioural and emotional problems persisting into childhood and adolescence
Walsh et al. [33]	Prospective cohort (US adolescents)	Prenatal depressive symptoms (with childhood abuse interaction)	Inflammatory activation (IL-6, CRP) and increased cortisol during mid-pregnancy influencing gestational age	Gestational age at birth; birth weight	Elevated prenatal depressive symptoms were associated with immune and HPA-axis dysregulation; inflammatory markers predicted shorter gestation, supporting biological embedding in utero
Wei et al. [34]	Prospective longitudinal birth cohort (GUSTO, Singapore)	Prenatal maternal depressive symptoms (EPDS at 26 weeks)	Sex-dependent cortical programming; proposed HPA-axis and inflammatory pathways	Cortical surface area; reward sensitivity at 6 years	Prenatal depressive symptoms were associated with sex-specific alterations in cortical surface area; reduced superior parietal lobe surface area mediated increased reward sensitivity in girls, supporting neurodevelopmental transmission pathway
Pariante [35]	Narrative review integrating human, animal and cellular models	Antenatal depression (major depressive disorder during pregnancy)	Proposed glucocorticoid receptor (GR) resistance and HPA-axis hyperactivity; increased inflammation; cortisol-mediated foetal programming; epigenetic modification of GR (NR3C1); altered oxytocin receptor (OXTR) expression	Maternal care behaviour; offspring emotional and behavioural problems; increased HPA-axis reactivity; epigenetic changes in stress-related genes	Antenatal depression associated with HPA-axis hyperactivity and inflammatory activation; evidence supports cortisol-mediated in-utero programming; increased GR methylation observed in offspring exposed to prenatal maternal depression, suggesting biological intergenerational transmission
Spry et al. [36]	Prospective intergenerational cohort (VIHCS & ATPG3), *N* = 756 women, 1231 infants	Persistent maternal mental health problems (adolescence & young adulthood); antenatal & postnatal depressive symptoms	Preconception exposure; antenatal (third trimester); postnatal (1 year). Mediation analysis examined in-utero and perinatal pathways	Infant emotional reactivity at 1 year (Short Temperament Scale for Toddlers)	Persistent preconception mental health problems doubled the odds of high infant emotional reactivity (adjusted OR 2.1). Total effect = 0.42 SD increase in reactivity; 88% of effect was direct (not mediated by antenatal or postnatal depression). Findings suggest intergenerational transmission processes begin before conception, with limited mediation via in-utero depressive exposure.
Davis & Narayan [37]	Narrative review integrating intergenerational and DOHaD frameworks	Prenatal depression, anxiety, PTSD, and stress physiology; maternal childhood adversity affecting prenatal mental health	In-utero transmission via HPA-axis dysregulation; placental CRH; cortisol exposure; stress-related neurobiological programming	Preterm birth; low birth weight; infant cortisol reactivity; negative emotionality; internalising symptoms; cognitive and executive function; altered brain connectivity	Synthesises evidence that prenatal maternal mental health problems are associated with altered foetal programming of stress physiology and brain development. Highlights placental CRH and maternal cortisol as key biological pathways of in-utero intergenerational transmission. Emphasises both risk transmission and potential resilience processes during pregnancy.
O’Donnell et al. [38]	Prospective longitudinal cohort (ALSPAC), *n* ≈ 7,944 mother–child pairs	Prenatal anxiety and depression	Mid–late pregnancy (18 & 32 weeks gestation); hypothesised foetal programming via in-utero mechanisms (independent of postnatal mood and paternal mood)	Persistent behavioural and emotional symptoms across childhood; predicted probability of mental disorder	Prenatal anxiety predicted persistently higher total SDQ scores from ages 4–13 years. Effects were confined to higher intercept (stable elevation over time), not slope, supporting programming hypothesis. Children exposed to high prenatal anxiety (top 15%) had ~12.3% risk of probable disorder vs 6.8% in comparison group (≈ twofold increase), independent of postnatal maternal mood, paternal mood, obstetric and psychosocial confounders. Maternal effects stronger than paternal prenatal mood, supporting intrauterine pathway.
Lewis et al. [26]	Narrative review grounded in Developmental Origins of Health and Disease (DOHaD) model	Antenatal maternal depression, anxiety, psychological stress	In-utero programming via placental function, altered glucocorticoid exposure, epigenetic modification (e.g. NR3C1 methylation), HPA-axis development	Emotional disorders, behavioural problems, ADHD symptoms, stress dysregulation in childhood and adolescence	Synthesised evidence supports a foetal programming pathway linking maternal prenatal distress to altered offspring stress biology and increased risk of emotional and behavioural disorders. Highlights placental glucocorticoid regulation and epigenetic mechanisms as key biological mediators of intergenerational transmission. Emphasises the perinatal period as a critical window for prevention
Robinson et al. [28]	Systematic mini-review of 13 longitudinal cohort studies (e.g. PREDO, ALSPAC, Generation R, MoBa, SLCD, US registry cohorts)	Maternal prenatal depression (diagnosed or symptom-based measures such as EPDS, CES-D, CIS-R)	In-utero programming mechanisms proposed: HPA-axis dysregulation, placental glucocorticoid sensitivity (11β-HSD2), altered placental gene expression, epigenetic modifications (e.g. NR3C1 methylation), inflammatory pathways	Internalising and externalising problems, ADHD, anxiety disorders, autism spectrum traits/disorder, adult depression	Majority of studies (10/13) supported prenatal-specific effects independent of postnatal depression. Evidence indicates gestation-week non-specific effects where repeatedly measured (e.g. PREDO). Findings support foetal programming pathways linking maternal prenatal depression to offspring neuropsychiatric vulnerability across childhood into adulthood.
Bleker et al. [27]	Multi-country (USA, UK, Canada, China, India; synthesis of 13 prospective cohorts)	Systematic review of prospective cohort studies	Prenatal depressive disorder or depressive symptoms (various validated scales, e.g. EPDS, BDI, CES-D, DSM-IV interviews)	Assessed across pregnancy (varied by study; mostly second and third trimester)	HPA-axis reactivity (salivary cortisol); autonomic nervous system reactivity (heart rate, blood pressure, respiratory sinus arrhythmia, salivary alpha-amylase)
Turk et al. [39]	*N* = 49 adult offspring (High anxiety *n* = 13; Low-to-medium *n* = 36)	State Trait Anxiety Inventory (STAI); high anxiety defined as >43 (≥75th percentile) at 12–22 weeks gestation	Resting-state functional MRI; ROI-to-ROI connectivity (32 regions); Network-Based Statistics; Graph theory metrics	High prenatal maternal anxiety associated with significantly weaker functional connectivity between medial prefrontal cortex (MPFC) and left inferior frontal gyrus (IFG); additional weaker connectivity between left lateral prefrontal cortex (LPFC) and left somatosensory motor gyrus (SMG). Effects predominantly left-lateralised. No significant differences in global network metrics. Prenatal anxiety explained ~18% of variance in MPFC–IFG connectivity.	Neurobiological foetal programming; long-term frontal network alteration
Chae et al. [40]	Prospective longitudinal Chilean cohort (*n* = 1273 mothers)	Maternal depressive symptom trajectories (CES-D)	Postnatal depression trajectories across childhood (no direct in-utero assessment)	Internalising, externalising and attention problems (adolescence & adulthood); cognitive functioning at adolescence	Chronic severe maternal depression was associated with higher internalising and externalising symptoms into adulthood and lower adolescent verbal and mathematical performance. However, exposure was not measured during pregnancy; therefore, evidence relates to postnatal intergenerational transmission rather than in-utero programming.
Haavik et al. [41]	Narrative review integrating human multigenerational family data and animal studies	Maternal genotype influencing serotonin production (TPH1 mutations); maternal genetic variation affecting thyroid hormones, folate metabolism, immune activation, and glucocorticoid exposure	Altered intrauterine biochemical environment via reduced maternal serotonin, hormonal dysregulation, micronutrient metabolism differences, cytokine signalling, and stress-axis programming	ADHD-related symptoms; broader neurodevelopmental vulnerability	Maternal TPH1 mutations associated with increased ADHD-related symptoms in offspring independent of child genotype; maternal genetic variation conceptualised as an ‘environmental factor’ shaping foetal neurodevelopment; supports in-utero programming as a mechanism contributing to intergenerational transmission of mental health risk
Waxler et al. [42]	Narrative review integrating human cohort and experimental evidence	Antenatal maternal depression	Elevated maternal cortisol and placental CRH during second and third trimesters; foetal HPA-axis programming; altered uterine blood flow; epigenetic modification (DNA methylation of stress-related genes)	Preterm birth; low birth weight; altered foetal growth; increased infant stress reactivity; dysregulated HPA-axis function; insecure attachment; later internalising and externalising problems	Antenatal depression associated with increased maternal cortisol and CRH exposure to the foetus, mediating shorter gestation and reduced foetal growth. Evidence supports in-utero stress programming of infant neuroendocrine function and temperament, with downstream risk for emotional and behavioural disorders. Review also highlights gene–environment interaction and epigenetic mechanisms in intergenerational transmission.
Glover [43]	Narrative review synthesising prospective human cohorts and animal studies	Maternal depression, anxiety and psychosocial stress during pregnancy	Foetal programming mechanisms proposed: altered placental 11β-HSD2 expression; foetal cortisol exposure; placental serotonin synthesis; inflammatory cytokines; epigenetic modification (e.g. glucocorticoid receptor methylation)	Increased risk of emotional problems, ADHD symptoms, conduct disorder, reduced cognitive performance; lower birthweight; preterm birth; altered HPA-axis regulation; shorter telomere length	Evidence from multiple perspective cohorts indicates prenatal stress contributes approximately 10–15% of attributable risk for childhood behavioural problems. Associations persist after adjusting for postnatal maternal mood and paternal mood in several studies. Animal and human data support causal intrauterine programming mechanisms involving placental glucocorticoid regulation and epigenetic modification.
Stürmlinger et al. [44]	Longitudinal cohort, German Family Panel (*N* = 582)	Prenatal depressive symptoms	Assessed during pregnancy (T0). Authors discuss biological in-utero transmission pathways including foetal programming and HPA-axis mechanisms (conceptual framing).	Child adjustment difficulties at age 3 (internalising & externalising symptoms); infant temperament (Year 1)	Prenatal depressive symptoms significantly predicted child adjustment difficulties at age 3. PD symptoms also predicted more difficult infant temperament and lower parental role satisfaction. However, infant temperament and parental role satisfaction did not mediate the association between prenatal depressive symptoms and child adjustment difficulties. Evidence supports direct prenatal transmission effects rather than full serial mediation.
Tuovinen et al. [30]	Narrative review of 8 prospective and clinical studies (2017–2019)	Prenatal depressive symptoms, prenatal stress, mood disorders	Pregnancy; discusses foetal programming mechanisms (placental epigenetic changes, glucocorticoid regulation, inflammatory pathways, HPA-axis alterations)	Neurocognitive development (IQ, language, motor development, developmental milestones)	Evidence was mixed and inconsistent. Most studies found associations attenuated after covariate adjustment. Only the Finnish PREDO cohort showed robust independent associations between prenatal depressive symptoms and developmental delay. The review concludes that current evidence does not consistently support an independent prenatal effect, though biological programming mechanisms are plausible. Larger and methodologically rigorous studies are needed.
Entringer et al. [45]	Narrative review synthesising human cohort and experimental studies	Maternal psychosocial stress, pregnancy anxiety, depression, biological stress markers (cortisol, placental CRH, inflammatory cytokines)	Maternal–placental–foetal (MPF) stress biology; HPA-axis activation; altered 11β-HSD2 activity; inflammatory pathways; telomere programming	Brain development (amygdala, hippocampus, cortical thickness), executive function, affective problems, metabolic dysfunction, immune alterations, telomere length	Evidence supports foetal programming model whereby prenatal stress exposure alters maternal–placental–foetal endocrine and immune processes, influencing offspring neurodevelopment and long-term physical and mental health risk. Early gestation appears particularly sensitive. Telomere biology proposed as a novel intergenerational mechanism. Effects not fully explained by adverse birth outcomes.
Kim et al. [46]	Prospective cohort (Bt20+, South Africa), *n* = 304 mother–child pairs	Prenatal stress (psychosocial adversity, violence, trauma exposure during apartheid)	Third trimester; cumulative stress score during pregnancy	Psychiatric morbidity (GHQ-28) at age 17–18; interaction with postnatal stress	No direct main effect of prenatal stress on psychiatric morbidity in late adolescence. However, significant stress-sensitisation effect: prenatal stress amplified the impact of later-life household trauma. Effects stronger among offspring of younger mothers. Social support did not buffer effects. Suggests in-utero stress exposure increases vulnerability to future stress rather than causing direct psychopathology.
Tirumalaraju et al. [29]	Systematic review and Bayesian meta-analysis of 6 prospective longitudinal birth cohorts (*n* ≈ 19,535 mother–child dyads)	Maternal antenatal and/or postnatal depression	Antenatal depression (during pregnancy) and postnatal depression (≤12 months postpartum); biological stress pathways (HPA-axis dysregulation, inflammation) discussed as potential in-utero mechanisms	Adolescent and adult depression (diagnosed via validated instruments and clinical interviews)	Maternal perinatal depression associated with 70% increased odds of offspring depression (pooled OR 1.70; 95% CI 1.06–2.65). Antenatal depression showed slightly stronger association (OR 1.78) than postnatal depression (OR 1.66). Findings support intergenerational transmission of depression risk, with potential in-utero biological programming pathways implicated.

### Interpretation in light of the conceptual framework

Viewed through the proposed conceptual framework, the findings support pregnancy as a potentially sensitive developmental period during which maternal mental health may shape the intrauterine environment and influence foetal neurodevelopment. The reviewed sources of evidence indicate several plausible pathways, including neuroendocrine, inflammatory, and epigenetic processes, through which prenatal psychological distress may be linked to offspring stress regulation, emotional processing, and cognitive functioning. These proposed biological processes provide plausible explanations for the observed associations but should not be interpreted as established causal pathways, given that most included evidence was observational. Evidence of timing effects, including trimester-specific associations and differences by exposure duration, further suggests that gestation may include biologically and developmentally sensitive periods. Although the direction of association was generally consistent across the included sources of evidence, variation in the magnitude and significance of reported effects likely reflects differences in maternal mental health measures, timing of exposure assessment, offspring outcome definitions, duration of follow-up, and population characteristics. These findings also suggest that potential biological programming processes may interact with postnatal caregiving environments and broader socioeconomic contexts, contributing to heterogeneity in developmental outcomes across studies.

These findings are broadly consistent with previous reviews linking prenatal maternal depression, anxiety, and stress to adverse offspring emotional, behavioural, neurodevelopmental, and psychiatric outcomes. However, unlike previous reviews, which have generally focused on specific mental health conditions, biological mechanisms, or individual developmental outcomes, the present review integrates evidence across multiple maternal mental health exposures, developmental outcomes, and proposed biological pathways within a unified framework of potential intergenerational and in-utero pathways. In addition, this review explicitly considers the broader implications for population health, health economics, and policy. At the same time, the evidence does not suggest that prenatal influences operate in isolation. Postnatal maternal mental health, caregiving environments, and broader socioeconomic conditions remain important and may amplify, attenuate, or mediate the associations between prenatal maternal mental health and offspring outcomes. These contextual influences are also likely to interact with biological programming processes, which may help explain the heterogeneity in offspring outcomes observed across studies despite similar prenatal exposures. From an intervention perspective, this suggests that strengthening postnatal maternal mental health and caregiving environments may help mitigate some of the longer-term effects of prenatal psychological distress, even when prenatal exposure has already occurred. Nevertheless, the persistence of associations into later childhood and adolescence indicates that prenatal exposure may contribute to longer-term developmental trajectories. This interpretation is consistent with life-course and developmental origins perspectives and highlights the importance of distinguishing prenatal from postnatal mechanisms where possible.

### Implications for health policy

From a health economics perspective, the findings highlight prenatal maternal mental health as a potentially important early-life exposure with long-run implications for human capital formation. Offspring mental health and cognitive development are closely linked to educational attainment, labour market participation, and productivity, yet these downstream consequences are rarely incorporated into economic evaluations of maternal mental health interventions. As a result, the broader societal benefits of improving antenatal mental health may be underestimated.

The predominance of observational evidence and the limited integration of economic outcomes also point to an important gap at the intersection of epidemiology and economics. Incorporating prenatal maternal mental health into economic models of intergenerational inequality, early-life investment, and prevention could strengthen the policy relevance of this literature. Pregnancy may represent a particularly important window for intervention, especially in contexts of socioeconomic disadvantage or heightened stress exposure, including disasters and other adverse events. These findings also support routine assessment of maternal mental health during antenatal care and timely referral to appropriate psychological and psychosocial support services as part of comprehensive maternal healthcare.

The limited representation of low- and middle-income countries also raises concerns regarding global relevance and equity. Maternal mental health burdens are often substantial in resource-constrained settings, yet evidence on association between prenatal maternal mental health and offspring outcomes, including potential in-utero pathways, remains sparse in these contexts. Expanding research beyond high-income countries is therefore important for informing globally relevant maternal and child health policy and for avoiding inappropriate extrapolation across markedly different institutional and social settings.

### Strengths and limitations

This review has several strengths. By adopting a scoping review approach, it was able to synthesise a heterogeneous and interdisciplinary body of literature that would not readily lend itself to meta-analysis because of variation in exposure definitions, outcome measures, study designs, and follow-up periods. The use of a PECO-informed framework and an explicit conceptual model also enabled a focused examination of prenatal exposures and postnatal offspring outcomes while avoiding overly causal interpretation.

Several limitations should also be acknowledged. First, the review was restricted to English-language, peer-reviewed publications, which may have excluded relevant evidence published in other languages or in the grey literature. Second, consistent with PRISMA-ScR guidance, no formal critical appraisal was undertaken; therefore, the methodological quality and risk of bias of the included sources of evidence were not systematically assessed. Third, substantial heterogeneity in exposure measurement, diagnostic instruments, outcome definitions, timing of assessment, and follow-up duration limits comparability across studies. Fourth, most included primary studies were observational, which constrains causal inference because shared genetic, environmental, and socioeconomic factors may contribute to the reported associations. In addition, because the review was restricted to peer-reviewed publications, the potential for publication bias cannot be excluded. The predominance of evidence from high-income countries also limits the generalisability of the findings to low- and middle-income settings. Finally, the inclusion of both primary studies and synthesis-based evidence may increase the risk of overlap in reported findings across the literature base. Future research would benefit from greater standardisation of maternal mental health measures and offspring outcome definitions, longer-term longitudinal follow-up, improved representation of low- and middle-income countries, and the integration of biological, psychosocial, and economic perspectives to strengthen understanding of the potential biological, psychosocial, and intergenerational pathways underlying these associations.

## Conclusions

This scoping review found that maternal mental health during pregnancy is frequently associated with offspring mental, cognitive, and psychological outcomes, with reported associations extending into later developmental stages. Taken together, the literature is consistent with prenatal maternal mental health being an important early-life exposure associated with later developmental trajectories. However, the available evidence does not establish causal in-utero transmission, and the relative contributions of potential intrauterine, postnatal, genetic, and shared familial mechanisms remain difficult to disentangle.

### Future directions

Future research should prioritise stronger causal identification strategies, improved longitudinal data linkage, clearer separation of prenatal and postnatal influences, and the incorporation of economic outcomes to better estimate the longer-term returns to antenatal mental health interventions. Greater standardisation of maternal mental health measures and offspring outcome assessments, together with longer-term follow-up across diverse populations, would strengthen the evidence base and improve comparability across studies. Framing maternal mental health during pregnancy as a potentially important intergenerational exposure, rather than only a short-term clinical concern, may help inform maternal mental health interventions, strengthen prenatal care policies, and improve long-term population health. Table 4 summarises the principal evidence gaps identified across the included sources of evidence together with their implications for future research, health economics, and policy.

**Table 4.** Key evidence gaps and implications for future research and policy.

Evidence gap identified	Summary of evidence from included sources of evidence (n = 20)	Implications for health economics and policy
Limited long-term follow-up into adulthood	While several prospective cohorts demonstrated associations between prenatal maternal depression, anxiety, or stress and offspring emotional, behavioural, neurocognitive and psychiatric outcomes, only a small subset extended follow-up into adulthood. Adult outcomes were primarily neurobiological (e.g. functional connectivity) or psychiatric risk, with few studies examining sustained functional or socioeconomic consequences.	Economic modelling of lifetime burden remains incomplete. Longitudinal linkage to labour market outcomes, healthcare utilisation, and productivity loss is required to quantify intergenerational cost transmission and justify investment in antenatal mental health interventions.
Mechanistic fragmentation across studies	Evidence converged on foetal programming pathways, including HPA-axis dysregulation, altered cortisol exposure, epigenetic modification, inflammatory processes, and structural and functional brain alterations (particularly prefrontal–limbic circuitry). However, studies often examined isolated mechanisms rather than integrated biological pathways.	Integrated biomarker–neuroimaging–clinical designs are needed to strengthen causal inference. Mechanistic clarity supports cost-effectiveness arguments for preventive screening and early intervention in pregnancy.
Underrepresentation of intergenerational transmission frameworks	Although several studies adopted developmental programming models, few explicitly modelled multigenerational transmission beyond mother–child dyads or examined persistence into the next reproductive generation.	Policies addressing maternal mental health should be framed as intergenerational human capital investments. Prevention during pregnancy may yield returns beyond one generation.
Inconsistent measurement of prenatal exposure	Studies varied in timing (early vs mid vs late pregnancy), measurement tools (self-report scales vs clinical diagnosis), and exposure definitions (state anxiety, trait anxiety, depression, cumulative adversity).	Standardised exposure measurement would improve comparability and allow meta-analytic cost projections. Routine antenatal screening protocols should adopt validated, harmonised tools.
Limited integration of biological and socioeconomic outcomes	Neuroimaging and biological studies identified structural and functional brain changes persisting into childhood and adulthood, yet few linked these alterations to educational attainment, occupational outcomes, or long-term economic participation.	Future research should integrate neurodevelopmental markers with education and employment trajectories to quantify downstream economic burden. This would strengthen arguments for universal perinatal mental health services.
Small sample sizes in mechanistic studies	Several neurobiological and imaging studies relied on modest cohort sizes, limiting power to detect sex-specific effects or interaction pathways.	Larger population-based cohorts are required to estimate population attributable fractions and inform national-level cost-benefit analyses.
Limited evaluation of preventive interventions	Most studies focused on observational associations rather than testing whether reducing maternal anxiety or depression during pregnancy alters offspring trajectories.	There is a need for economic evaluation of antenatal mental health prevention programs, including universal screening, stepped-care models, and early psychosocial interventions.
Underestimation of broader societal costs	Evidence consistently showed associations between prenatal maternal mental health and offspring behavioural dysregulation, executive dysfunction, psychiatric risk, and altered brain connectivity. However, few studies quantified healthcare, educational, or productivity costs associated with these outcomes.	Health economic frameworks should incorporate lifetime mental health burden, educational remediation costs, and productivity losses. Early investment in maternal mental health may generate long-term fiscal savings.

## Supplementary Material

Supplementary Document.docx

Prisma Scr Checklist.docx

## Data Availability

The data supporting the findings of this study are derived from publicly available published studies, which are cited within the article. Extracted data are available from the corresponding author upon reasonable request.
