# Supplementary material for: Maternal mental health and its intergenerational transmission in-utero: a scoping review
Source: Glob Health Action. 2026 Oct 22;19(1):2750711. doi: 10.1080/16549716.2026.2750711 (PMC13700464; doi:10.1080/16549716.2026.2750711)
Supplement: Supplementary file 1 [file ZGHA_A_2750711_SM6252.docx]

**Preferred Reporting Items for Systematic reviews and Meta-Analyses extension for Scoping Reviews (PRISMA-ScR) Checklist**

| **SECTION** | **ITEM** | **PRISMA-ScR CHECKLIST ITEM** | **REPORTED ON PAGE #** |
| --- | --- | --- | --- |
| **TITLE** | | | |
| Title | 1 | Identify the report as a scoping review. | This report is a scoping review. |
| **ABSTRACT** | | | |
| Structured summary | 2 | Provide a structured summary that includes (as applicable): background, objectives, eligibility criteria, sources of evidence, charting methods, results, and conclusions that relate to the review questions and objectives. | A structured abstract is provided, including background, objectives, eligibility criteria, information sources, data charting methods, results, and conclusions, consistent with PRISMA-ScR guidance. |
| **INTRODUCTION** | | | |
| Rationale | 3 | Describe the rationale for the review in the context of what is already known. Explain why the review questions/objectives lend themselves to a scoping review approach. | The rationale for this review is described in the Introduction, highlighting the growing but fragmented evidence on maternal mental health during pregnancy and its in-utero intergenerational transmission to offspring outcomes. A scoping review approach was adopted to map the breadth, characteristics, and gaps in the literature, given heterogeneity in study designs, exposures, outcomes, and follow-up periods. |
| Objectives | 4 | Provide an explicit statement of the questions and objectives being addressed with reference to their key elements (e.g., population or participants, concepts, and context) or other relevant key elements used to conceptualize the review questions and/or objectives. | The review objectives are explicitly stated with reference to key elements, including the population (pregnant women and their offspring), the exposure (maternal mental health during pregnancy), and the outcomes (offspring mental, cognitive, and psychological outcomes). |
| **METHODS** | | | |
| Protocol and registration | 5 | Indicate whether a review protocol exists; state if and where it can be accessed (e.g., a Web address); and if available, provide registration information, including the registration number. | No formal review protocol was registered for this scoping review. |
| Eligibility criteria | 6 | Specify characteristics of the sources of evidence used as eligibility criteria (e.g., years considered, language, and publication status), and provide a rationale. | Eligibility criteria included peer-reviewed studies published in English that examined maternal mental health exposures during pregnancy and reported postnatal offspring mental, cognitive, or psychological outcomes. Studies focusing exclusively on birth outcomes or purely mechanistic biological outcomes without postnatal child outcomes were excluded. No restrictions were placed on study design. |
| Information sources* | 7 | Describe all information sources in the search (e.g., databases with dates of coverage and contact with authors to identify additional sources), as well as the date the most recent search was executed. | The following electronic databases were searched: MEDLINE, Scopus, PsycInfo, CINAHL, and Embase. The most recent search was conducted in February 2026 |
| Search | 8 | Present the full electronic search strategy for at least 1 database, including any limits used, such that it could be repeated. | The full electronic search strategy for at least one database is provided in the Supplementary Material. |
| Selection of sources of evidence† | 9 | State the process for selecting sources of evidence (i.e., screening and eligibility) included in the scoping review. | Sources of evidence were selected through a multi-stage screening process involving title and abstract screening followed by full-text assessment. Screening focused on relevance to prenatal maternal mental health exposure, explicit in-utero mechanisms, and postnatal offspring outcomes. |
| Data charting process‡ | 10 | Describe the methods of charting data from the included sources of evidence (e.g., calibrated forms or forms that have been tested by the team before their use, and whether data charting was done independently or in duplicate) and any processes for obtaining and confirming data from investigators. | Data were charted using a structured extraction framework developed for this review. Information was extracted on study characteristics, prenatal maternal mental health exposures, in-utero mechanisms, offspring outcomes, and key findings relevant to intergenerational transmission. |
| Data items | 11 | List and define all variables for which data were sought and any assumptions and simplifications made. | Data items included author and year, country or cohort, study design, prenatal maternal mental health exposure, timing and in-utero mechanisms, offspring mental, cognitive, or psychological outcomes, and key contributions to intergenerational transmission. |
| Critical appraisal of individual sources of evidence§ | 12 | If done, provide a rationale for conducting a critical appraisal of included sources of evidence; describe the methods used and how this information was used in any data synthesis (if appropriate). | Consistent with PRISMA-ScR guidance, no formal critical appraisal of individual sources of evidence was conducted, as the purpose of this review was to map the evidence rather than to assess study quality or estimate effects. |
| Synthesis of results | 13 | Describe the methods of handling and summarizing the data that were charted. | Charted data were synthesised narratively and thematically, with studies grouped according to exposure type, timing, and offspring outcomes. |
| **RESULTS** | | | |
| Selection of sources of evidence | 14 | Give numbers of sources of evidence screened, assessed for eligibility, and included in the review, with reasons for exclusions at each stage, ideally using a flow diagram. | A total of 6,930 records were identified across databases. After deduplication, 3,845 records remained for screening. Following sequential screening and eligibility assessment, 50 studies were identified as relevant, and 20 core studies were included in the final scoping review. The selection process is summarised using a PRISMA-ScR flow diagram. |
| Characteristics of sources of evidence | 15 | For each source of evidence, present characteristics for which data were charted and provide the citations. | Characteristics of the included sources of evidence, including study design, exposure, outcomes, and key findings, are presented in Table 3 |
| Critical appraisal within sources of evidence | 16 | If done, present data on critical appraisal of included sources of evidence (see item 12). | Not applicable, as no formal critical appraisal was conducted. |
| Results of individual sources of evidence | 17 | For each included source of evidence, present the relevant data that were charted that relate to the review questions and objectives. | Relevant data from each included source of evidence are presented in the Results section and summarised in Table 2 |
| Synthesis of results | 18 | Summarize and/or present the charting results as they relate to the review questions and objectives. | The results are synthesised thematically in relation to prenatal maternal mental health exposures, in-utero mechanisms, and offspring outcomes, aligned with the review objectives. |
| **DISCUSSION** | | | |
| Summary of evidence | 19 | Summarize the main results (including an overview of concepts, themes, and types of evidence available), link to the review questions and objectives, and consider the relevance to key groups. | The main findings of the review are summarised in relation to the review objectives, highlighting key concepts, themes, and evidence gaps in the literature on in-utero intergenerational transmission of maternal mental health. |
| Limitations | 20 | Discuss the limitations of the scoping review process. | Limitations of the scoping review include restriction to English-language publications and the absence of formal quality appraisal, consistent with the objectives of evidence mapping. |
| Conclusions | 21 | Provide a general interpretation of the results with respect to the review questions and objectives, as well as potential implications and/or next steps. | The review provides an overview of existing evidence on prenatal maternal mental health and its intergenerational transmission, identifies important gaps, and outlines implications for future research and policy. |
| **FUNDING** | | | |
| Funding | 22 | Describe sources of funding for the included sources of evidence, as well as sources of funding for the scoping review. Describe the role of the funders of the scoping review. | This scoping review received no specific external funding. The funders had no role in the design, conduct, or reporting of the review. |

JBI = Joanna Briggs Institute; PRISMA-ScR = Preferred Reporting Items for Systematic reviews and Meta-Analyses extension for Scoping Reviews.

* Where *sources of evidence* (see second footnote) are compiled from, such as bibliographic databases, social media platforms, and Web sites.

† A more inclusive/heterogeneous term used to account for the different types of evidence or data sources (e.g., quantitative and/or qualitative research, expert opinion, and policy documents) that may be eligible in a scoping review as opposed to only studies. This is not to be confused with *information sources* (see first footnote).

‡ The frameworks by Arksey and O’Malley (6) and Levac and colleagues (7) and the JBI guidance (4, 5) refer to the process of data extraction in a scoping review as data charting*.*

§ The process of systematically examining research evidence to assess its validity, results, and relevance before using it to inform a decision. This term is used for items 12 and 19 instead of "risk of bias" (which is more applicable to systematic reviews of interventions) to include and acknowledge the various sources of evidence that may be used in a scoping review (e.g., quantitative and/or qualitative research, expert opinion, and policy document).

*From:* Tricco AC, Lillie E, Zarin W, O'Brien KK, Colquhoun H, Levac D, et al. PRISMA Extension for Scoping Reviews (PRISMAScR): Checklist and Explanation. Ann Intern Med. 2018;169:467–473. [doi: 10.7326/M18-0850](http://annals.org/aim/fullarticle/2700389/prisma-extension-scoping-reviews-prisma-scr-checklist-explanation).
