# Supplementary material for: Maternal mental health and its intergenerational transmission in-utero: a scoping review
Source: Glob Health Action. 2026 Oct 22;19(1):2750711. doi: 10.1080/16549716.2026.2750711 (PMC13700464; doi:10.1080/16549716.2026.2750711)
Supplement: Supplementary file 2 [file ZGHA_A_2750711_SM6253.docx]

Supplementary File S1-S5

| **S1** | | |
| --- | --- | --- |
| **Line** | **Search terms (Ovid MEDLINE syntax)** | **Records retrieved** |
| 1 | (pregnan* or prenatal or antenatal or perinatal or "in utero").mp. | 1,217,173 |
| 2 | (depress* or anxi* or distress or "mental health" or "psychological stress").mp. | 1,143,559 |
| 3 | (intergenerational or transmission or "fetal programming" or "prenatal programming" or DOHaD).mp. | 583,172 |
| 4 | (offspring or child* or infant* or neurodevelopment*).mp. | 3,453,209 |
| 5 | 1 AND 2 AND 3 AND 4 | **1,665** |

| **S2** | | |
| --- | --- | --- |
| **Line** | **Search terms (Scopus)** | **Records retrieved** |
| 1 | TITLE-ABS-KEY((pregnan* OR prenatal OR antenatal OR perinatal OR "in utero") AND (depress* OR anxi* OR distress OR "mental health" OR "psychological stress") AND (intergenerational OR transmission OR "fetal programming" OR "foetal programming" OR "prenatal programming" OR DOHaD OR "developmental origins") AND (offspring OR child* OR infant* OR neonat* OR neurodevelopment*)) | 3121 |
| 2 | Filters applied (if any): document type, language, source type | 1970 |

| **S3** | | |
| --- | --- | --- |
| **Line** | **Search terms (PSYCINFO)** | **Records retrieved** |
| 1 | (pregnan* OR prenatal OR antenatal OR perinatal OR "in utero").mp. | 101,871 |
| 2 | (depress* OR anxi* OR distress OR "mental health" OR "psychological stress").mp. | 965,727 |
| 3 | (intergenerational OR transmission OR "fetal programming" OR "prenatal programming" OR DOHaD).mp. | 74,561 |
| 4 | (offspring OR child* OR infant* OR neurodevelopment*).mp. | 1,055,581 |
| 5 | 1 AND 2 AND 3 AND 4 | **828** |
| 6 | Limit 5 to full text, journals, human, English language, abstracts | **60** |

| **S4** | | |
| --- | --- | --- |
| **Line** | **Search terms (CINAHL)** | **Records retrieved** |
| 1 | ((MH "Pregnancy+" OR MH "Prenatal Care+" OR pregnan* OR prenatal OR antenatal OR perinatal OR "in utero") AND (MH "Mental Health+" OR MH "Depression+" OR MH "Anxiety+" OR MH "Stress, Psychological+" OR depress* OR anxi* OR distress OR "mental health" OR "psychological stress") AND (intergenerational OR transmission OR "fetal programming" OR "foetal programming" OR "prenatal programming" OR DOHaD OR "developmental origins") AND (MH "Child+" OR MH "Infant+" OR MH "Child Development+" OR offspring OR child* OR infant* OR neonat* OR neurodevelopment*)) | **589** |

| **S5** | | |
| --- | --- | --- |
| **Line** | **Search terms (Embase.com syntax)** | **Records retrieved** |
| 1 | 'pregnancy'/exp OR 'pregnancy' OR 'pregnant woman'/exp OR 'pregnant woman' OR pregnan*:ti,ab,kw OR prenatal:ti,ab,kw OR antenatal:ti,ab,kw OR perinatal:ti,ab,kw OR 'in utero':ti,ab,kw | 1,543,212 |
| 2 | 'maternal depression'/exp OR 'depression'/exp OR 'anxiety'/exp OR 'psychological stress'/exp OR 'mental health'/exp OR depress*:ti,ab,kw OR anxi*:ti,ab,kw OR distress:ti,ab,kw OR 'mental health':ti,ab,kw OR 'psychological stress':ti,ab,kw | 2,138,753 |
| 3 | 'intergenerational relation' OR intergenerational:ti,ab,kw OR transmission:ti,ab,kw OR 'fetal programming':ti,ab,kw OR 'foetal programming':ti,ab,kw OR 'prenatal programming':ti,ab,kw OR dohad:ti,ab,kw OR 'developmental origins':ti,ab,kw | 624,464 |
| 4 | 'offspring'/exp OR 'child'/exp OR 'infant'/exp OR 'neurodevelopment'/exp OR offspring:ti,ab,kw OR child*:ti,ab,kw OR infant*:ti,ab,kw OR neonat*:ti,ab,kw OR neurodevelopment*:ti,ab,kw | 4,925,501 |
| 5 | **#1 AND #2 AND #3 AND #4** | **2,646** |
